# A Rare Twist: Case Study on Gastrohepatic Volvulus

**DOI:** 10.7759/cureus.111578

**Published:** 2026-06-26

**Authors:** Michael Ashley, Stephanie Nagy, David Kosoy

**Affiliations:** 1 Radiology, Aventura Hospital and Medical Center, Aventura, USA; 2 Radiology, Nova Southeastern University Dr. Kiran C. Patel College Of Osteopathic Medicine, Fort Lauderdale, USA

**Keywords:** adult intestinal malrotation, gastric volvulus, gastrohepatic volvulus, gastrointestinal malrotation, hepatic torsion

## Abstract

A gastric volvulus occurs when the stomach rotates around itself, creating a closed-loop obstruction. This can result in a life-threatening condition in which supporting vasculature can be obstructed and lead to ischemia, necrosis, and perforation. A gastrohepatic volvulus is an exceedingly rare type of gastric volvulus in which the stomach rotates in relation to the liver, specifically around the gastrohepatic ligament.

We present a case of a 39-year-old female with a history of congenital malrotation and developmental delay who presented to the emergency room for nausea and emesis. Physical examination revealed a distended and tense abdomen. A CT scan was completed, revealing abnormal positioning of a distended stomach located in the right upper quadrant and the liver located in the left upper quadrant. An emergent exploratory laparotomy revealed complete gastrohepatic volvulus. The stomach and liver were both found to have visible signs of ischemia with dusky discoloration of both organs. After manual detorsion, adequate blood flow was re-established, as evidenced by the return of normal coloration.

Gastrohepatic volvulus represents an exceedingly rare and previously unreported variant of gastric volvulus. This case highlights the potential for atypical anatomical presentations to mimic other conditions, such as heterotaxy syndrome. It underscores the critical importance of maintaining a high index of suspicion in patients with acute abdominal symptoms and abnormal imaging findings. Prompt recognition, a multidisciplinary approach, and emergent surgical intervention are essential to prevent irreversible ischemic injury and associated complications.

## Introduction

Gastric volvulus is a rare presentation that is uncommonly reported in the literature. It occurs when the stomach rotates at least 180 degrees along either the transverse or longitudinal axis. It is classified into two subtypes based on the axis of rotation: either organoaxial volvulus with rotation around the longitudinal axis or mesenteroaxial volvulus around the transverse axis [1]. Gastric volvulus typically occurs in isolation; however, in extremely rare cases, other structures can be impacted, including the spleen and duodenum.

Primary gastric volvulus arises from structural defects of the gastric ligaments, including agenesis, elongation, or disruption caused by neoplasia, adhesions, or skeletal deformity. Secondary gastric volvulus, which accounts for two-thirds of all cases, results from underlying anatomic abnormalities such as paraesophageal hernia, diaphragmatic hernia, diaphragmatic eventration, phrenic nerve paralysis, and wandering spleen, as well as structural anomalies of adjacent organs [2].

This condition is most commonly seen in patients between 65 and 75 years old [3,4]. Due to the rarity of the condition, the exact incidence cannot be determined. Patients with an acute gastric volvulus typically present with the triad of acute epigastric pain, retching, and the inability to pass a nasogastric tube, termed Borchardt's triad. If it progresses without identification, it can lead to septic shock due to necrosis. Patients with chronic gastric volvulus experience non-specific symptoms of upper abdominal pain, nausea, dysphagia, early satiety, vomiting, and hiccups [1]. If not treated early, mortality rates are estimated to range between 30 and 50%, indicating the importance of timely identification and management [1].

Hepatic torsion is an even more rare condition with only a limited number of case reports existing in the literature, occurring primarily in those with congenital accessory hepatic lobes (AHL) [5]. While the overwhelming majority of AHL are asymptomatic, they may rarely undergo isolated torsion of their supplying vascular pedicle. The contemporaneous occurrence of gastric and hepatic volvulus is exceptionally uncommon and may present with overlapping symptoms. To our knowledge, no reported cases in the literature document concurrent gastric and hepatic volvulus. As such, this condition may be overlooked.

This paper presents the novel case of a 39-year-old woman with congenital malrotation and developmental delay who presented to the emergency room for nausea and emesis, determined to be the cause of a gastrohepatic volvulus requiring surgical detorsion. There are a few case studies in the literature describing gastric volvulus involving adjacent organs, but none have reported concurrent gastric and complete hepatic volvulus. This case contributes a significant finding to the growing body of evidence regarding the wide variety of presentations of gastric volvulus.

## Case presentation

A 39-year-old woman with a past medical history of congenital malrotation, gastritis, and a developmental delay, non-verbal and non-ambulatory at baseline, presented to the emergency department (ED) with progressively worsening nausea and emesis beginning 24 hours prior to presentation. Her only medication was omeprazole for gastritis. Due to her non-verbal status at baseline, further details about her current illness were unable to be obtained.

In the ED, the patient’s vital signs were significant for tachycardia at 125. The remainder of the patients' vital signs at the time of presentation were within normal limits. Initial physical examination revealed diffuse abdominal tenderness. However, upon re-evaluation by the surgery service, the patient had significant abdominal distension and involuntary guarding. Initial laboratory results were significant for leukocytosis, elevated alkaline phosphatase, transaminitis, bilirubinemia, and lactic acidosis (Tables 1-2). These laboratory findings suggested ongoing ischemia and developing liver injury.

**Table 1:** Laboratory results for complete blood count.

Labs	Findings	Normal range
Red blood cell	5.14 10^6^ µL	3.93-5.22 10^6^ µL
White blood cell	12.0 10^3^ µL	4-10.5 10^3^ µL
Hematocrit	47.6%	42-52%
Hemoglobin	16.1 g/dL	14-18 g/dL
Platelets	326 10^3^ µL	150-400 10^3^ µL
Segmented neutrophils	93.6%	34.0-71.1%
Mean corpuscular hemoglobin	31.5 pg/cell	25.6-32.2 pg/cell
Mean corpuscular volume	92.6 fL	80-100 fL
Red blood cell distribution	13.5%	11.7-14.4%
Basophils	0.2%	0.1-1.2%
Eosinophils	0%	0.7-5.8%
Lymphocytes	3.9%	19.3-51.7%
Monocytes	2.1%	4.7-12.5%
Granulocytes	0.2%	0-0.4%

**Table 2:** Laboratory results for complete metabolic panel.

Labs	Findings	Normal range
Alanine aminotransferase	237 U/L	10-40 U/L
Aspartate aminotransferase	226 U/L	10-60 U/L
Alkaline phosphatase	156 U/L	20-30 U/L
Bilirubin	1.6 mg/dL	0.1-1.2 mg/dL
Calcium	10.2 mg/dL	8.4-10.2 mg/dL
Carbon dioxide	20 mmol/L	19-34 mmol/L
Chloride	103 mEq/L	95-110 mEq/L
Creatinine	0.65 mg/dL	0.43-1.13 mg/dL
Estimated glomerular filtration rate	90 ml/min/1.73m^2^	>90 ml/min/1.73m^2^
Glucose	172 mg/dL	70-110 mg/dL
Lipase	64 U/L	23-300 U/L
Albumin	5.3 g/dL	3.2-5.0 g/dL
Potassium	4.2 mEq/L	3.7-5.2 mEq/L
Sodium	145 mEq/L	135-145 mEq/L
Blood urea nitrogen	18 mg/dL	6-22 mg/dL
Lactic acid	8.8 mmol/L	0.4-2 mmol/L

Imaging with non-contrast-enhanced computed tomography (NECT), ordered per the ED, revealed heterotopic positioning of the stomach and liver in the right upper quadrant and the left upper quadrant, respectively. Contrast-enhanced computed tomography (CECT) was not ordered. In addition, there was marked gastric distension and swirling of the mesentery supplying the stomach, raising concern for an internal hernia resulting in gastric obstruction (Figures 1-2).

**Figure 1:**
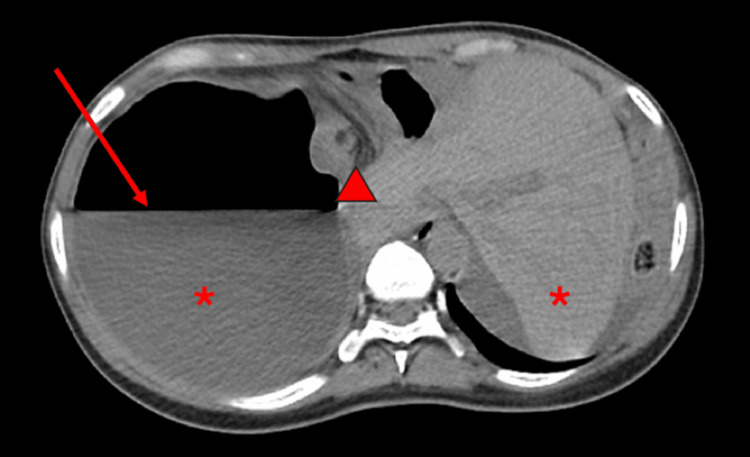
Axial non-contrast-enhanced computed tomography (NECT) of the upper abdomen at presentation. There is a heterotopic location of the stomach and liver, located in the right upper quadrant and left upper quadrant, respectively (asterisks). There is a subtle swirling of the vascular pedicle adjacent to the lesser curvature of the stomach (arrowhead) and gastric distension (arrow), concerning for an internal hernia with gastric obstruction. NECT: non-contrast-enhanced computed tomography.

**Figure 2:**
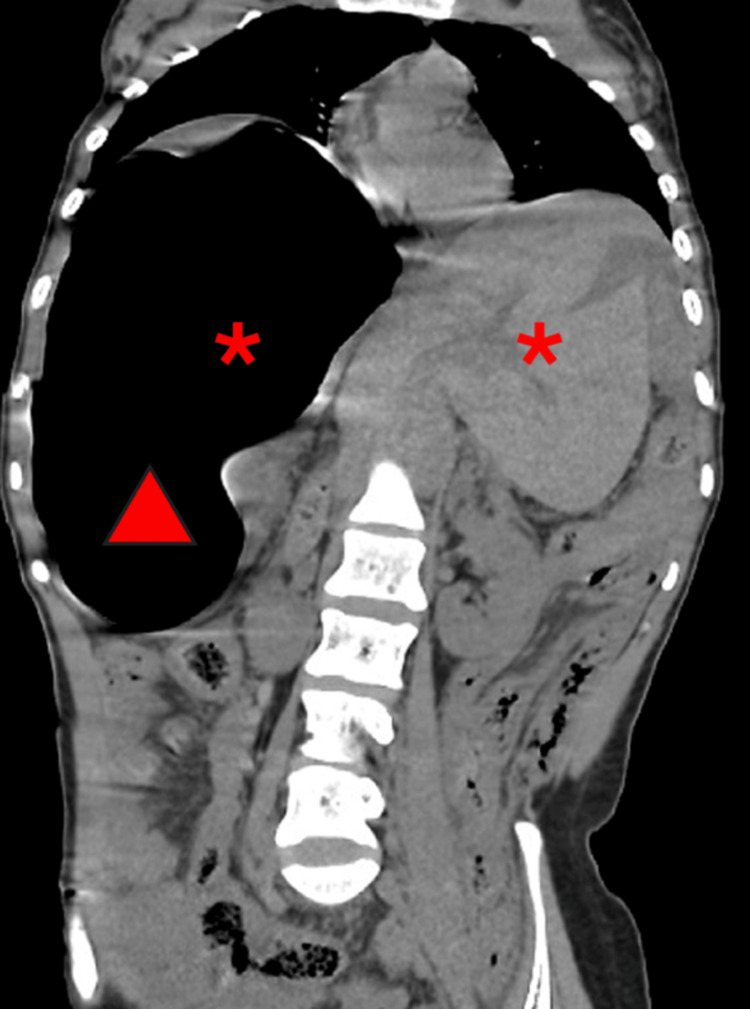
Coronal NECT of the abdomen and pelvis redemonstrates heterotopic positioning of the stomach and liver (asterisks) and marked gastric distension (arrowhead). NECT: non-contrast-enhanced computed tomography.

The patient was urgently taken to the operating room (OR) for an exploratory laparotomy. In the OR, the stomach and liver were confirmed to be in heterotopic positions. Upon further exploration, a complete hepatogastric volvulus was identified, with both the stomach and liver appearing ecchymotic and purplish in color, indicative of ischemia. Upon manual detorsion, there was a prompt return of normal coloration to the stomach and liver. Due to the complexity of the situation, images were not obtained during the procedure. Gastropexy was performed, and a gastrostomy tube was placed. The patient was then transferred to the intensive care unit.

The patient’s postoperative course was complicated by a deep vein thrombosis (DVT) and portal vein thrombosis (PVT) (Figure 3). The PVT was presumed to be as a result of the patient's hepatic volvulus, leading to stasis of the portal blood supply to the liver and subsequent thrombus formation. Further workup for an underlying hypercoagulable state was not pursued. Her clinical course stabilized, and she was discharged to a long-term care facility after 13 days. She was discharged on full-dose anticoagulation for treatment of her DVT and PVT.

**Figure 3:**
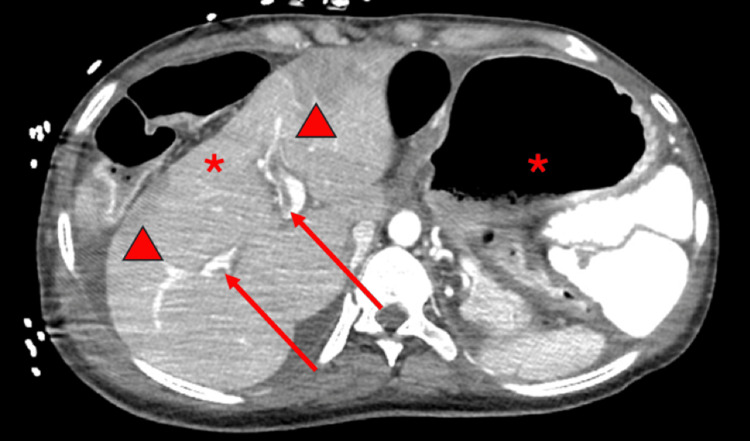
Postoperative axial CECT of the upper abdomen. There is now an orthotopic arrangement of the stomach and liver after surgical correction (asterisks). There is interval development of PVT within the right and left intrahepatic portal veins (arrows). The wedge-shaped areas of hypoattenuation along the periphery of the liver (arrowheads) are likely a result of altered hepatic perfusion from PVT and/or hepatic infarct. CECT: contrast-enhanced computed tomography; PVT: portal vein thrombosis.

## Discussion

Gastric volvulus is a rare and complex disorder that, if not promptly diagnosed and treated, carries a significant risk of morbidity and mortality as a result of ischemia. While some cases of acute or chronic gastric volvulus may be managed conservatively with gastric decompression, this case highlights the necessity of emergent surgical intervention in patients who are at risk of organ ischemia and medical decompensation. Mortality rates can range from 30% to 50% [6]. Gastric volvulus may rarely involve other abdominal viscera such as the duodenum, pancreas, or, as in this case, the liver. While 70% of gastric volvulus patients present with the classic Borchardt triad, the remaining patients may have a wide, non-specific history and presentation, which typically makes imaging the mainstay of diagnosis. On imaging, the diagnosis can be made with CT or an upper gastrointestinal series with oral contrast [1].

Due to overlapping imaging characteristics, the radiologic differential diagnosis for gastric volvulus includes large hiatal hernia, causes of gastric distension/gastric outlet syndrome (e.g., neoplasm and gastroparesis), and gastric or esophageal diverticula. In this case, due to the heterotopic locations of the stomach and liver, a unique differential to be considered is situs inversus or heterotaxy syndrome: a rare congenital disorder characterized by abnormal configuration of thoracic and abdominal viscera upon the left and right axis [7]. The patient in this case did have a history of congenital midgut malrotation surgically corrected in infancy, highlighting the importance of obtaining an accurate medical and surgical history in determining predisposing risk factors. This patient likely had a persistent internal hernia, which led to the atypical presentation and gastrohepatic volvulus. Other important predisposing risk factors for gastric volvulus include wandering spleen syndrome or prior gastric or bariatric surgeries [8,9].

Upon presentation, the patient in this case was found to be tachycardic, although without overt signs of shock. However, laboratory analysis was consistent with developing end-organ ischemia and hepatic injury with lactic acidosis, leukocytosis, and transaminitis. This case highlights the pivotal need for early identification and intervention in order to prevent continued end-organ ischemia, thus resulting in hemodynamic compromise and shock. As described, acute gastric volvulus and its variants may have a nebulous presentation, underscoring the importance of utilizing both the available clinical data and a multidisciplinary approach to achieve an accurate diagnosis and the best patient outcomes.

Initial management for acute gastric volvulus is achieved by gastric decompression with an enteric tube. Definitive treatment is done surgically with manual detorsion, assessment of gastric and associated abdominal viscera viability with resection as needed for necrosis, followed by gastropexy to prevent reoccurrence [10].

## Conclusions

This case identifies gastrohepatic volvulus as a rare variant of gastric volvulus. Gastrohepatic volvulus has been infrequently presented in the literature, which warrants further evaluation of its cause and should be considered when evaluating a patient with non-specific gastrointestinal complaints. Early recognition and timely surgical intervention are critical to preventing irreversible ischemic injury and reducing the risk of life-threatening complications.
